# Integrated Use of Late Gadolinium Enhancement and Left Ventricular Global Longitudinal Strain in Hypertrophic Cardiomyopathy

**DOI:** 10.1016/j.jacasi.2025.07.026

**Published:** 2026-02-03

**Authors:** Soongu Kwak, Jihoon Kim, Min-Ha Jeong, Chan Soon Park, Hyun-Jung Lee, Jun-Bean Park, Seung-Pyo Lee, Yoon Seong Lee, Eun-Ah Park, Whal Lee, Yong-Jin Kim, Hyung-Kwan Kim, Sang-Chol Lee

**Affiliations:** a Department of Internal Medicine, Seoul National University College of Medicine, Seoul, Republic of Korea; b Division of Cardiology, Cardiovascular Center, Seoul National University Hospital, Seoul, Republic of Korea; c Division of Cardiology, Department of Medicine, Samsung Medical Center, Heart Vascular Stroke Institute, Sungkyunkwan University School of Medicine, Seoul, Republic of Korea; d Department of Radiology, Seoul National University College of Medicine, Seoul, Republic of Korea

**Keywords:** cardiomyopathy, global longitudinal strain, hypertrophic, magnetic resonance imaging

## Abstract

**Background:**

Late gadolinium enhancement (LGE) and left ventricular global longitudinal strain (LV-GLS) are structural and functional left ventricular remodeling markers in hypertrophic cardiomyopathy (HCM).

**Objectives:**

This study investigated the prognostic value of integrating both markers for risk stratification in HCM.

**Methods:**

Consecutive patients with HCM who underwent CMR between 2008 and 2020 at 2 tertiary hospitals were retrospectively analyzed. LGE% and LV-GLS were measured, with LV-GLS reported as an absolute value. The primary outcome was cardiovascular (CV) events: CV death, sudden cardiac death–related events, and heart failure hospitalization.

**Results:**

Among 652 patients with HCM (median age 56 years; 74% [481 of 652] male), median LGE% and LV-GLS were 4.2% and 14.3%, respectively. During the median 7.4-year follow-up (Q1-Q3: 3.4-10.3 years), 59 patients (9.0%) experienced CV events. Higher LGE% and lower LV-GLS were associated with increased CV events (per 1% LGE% increase, adjusted HR [aHR]: 1.04 [95% CI: 1.01-1.06; *P* = 0.005]; per 1% LV-GLS decrease, aHR: 1.08 [95% CI 1.00-1.15; *P* = 0.038]). When analyzing the 4 groups stratified according to LGE% and LV-GLS, patients with both high LGE% (>4.2%) and low LV-GLS (<14.3%) had the highest incidence of CV events (aHR: 2.88; 95% CI: 1.21-6.85; *P* = 0.017). Mediation analysis showed a significant indirect effect of LGE% on CV events through LV-GLS. Sudden cardiac death–related events were most frequent in patients with both high LGE and low LV-GLS.

**Conclusions:**

LGE and LV-GLS provide complementary prognostic information in HCM. Patients with both increased LGE and decreased LV-GLS are at particularly high risk, highlighting the value of integrating both biomarkers for risk stratification.

Hypertrophic cardiomyopathy (HCM) is a prevalent inheritable cardiac condition associated with a significant risk of adverse cardiovascular (CV) events, including heart failure and sudden cardiac death (SCD).^1^^,^^2^ Accurate risk prediction in patients with HCM is crucial for optimizing prevention and treatment strategies.^3^^,^^4^ Although recent advances in cardiac imaging have introduced novel markers of myocardial remodeling and functional impairment, their integration into clinical practice remains an ongoing effort.^5^

Cardiovascular magnetic resonance (CMR) provides excellent soft tissue characterization, allowing for the assessment of replacement myocardial fibrosis through late gadolinium enhancement (LGE). Studies have established the extent of LGE as a noninvasive marker associated with increased adverse CV and SCD risk.^6^, ^7^, ^8^, ^9^, ^10^, ^11^, ^12^, ^13^ Moreover, myocardial fibrosis, as assessed by LGE, is closely associated with left ventricular (LV) decompensation, acting as a primary driver for progressive LV systolic dysfunction and end-stage heart failure.^14^, ^15^, ^16^, ^17^, ^18^, ^19^ LV global longitudinal strain (LV-GLS), a sensitive marker for detecting subclinical LV systolic dysfunction, has recently emerged as a powerful predictor of SCD in patients with HCM,^20^, ^21^, ^22^ even with preserved LV ejection fraction.^23^ Therefore, the assessment of both LGE and LV-GLS may offer valuable insights into the risk stratification of patients with HCM based on structural damage and functional impairment.

The aim of the current study was to investigate whether the integrated use of LGE and LV-GLS is clinically valuable for predicting CV events in patients with HCM. In addition, the potential mediating role of LV systolic function, measured by using LV-GLS, was explored in the association between LGE and CV events.

## Methods

### Cohort Characteristics

The study population included consecutive patients diagnosed with HCM from 2 tertiary university hospitals (Seoul National University Hospital and Samsung Medical Center) who underwent both CMR and echocardiography at the time of HCM diagnosis between 2008 and 2020. HCM was defined as maximal LV wall thickness ≥15 mm or ≥13 mm in individuals with a family history of HCM.^3^ Exclusion criteria were as follows: congenital heart disease, significant valvular heart disease (moderate or greater mitral and/or aortic regurgitation), end-stage renal disease, and infiltrative cardiomyopathy (ie, cardiac amyloidosis, Fabry disease, mitochondrial cardiomyopathy). In addition, those with an implantable cardioverter-defibrillator (ICD) at baseline or a history of aborted SCD were excluded. Patients with inadequate echocardiographic image quality for LV-GLS measurement were also excluded.

This study adhered to the principles of the Declaration of Helsinki and was approved by the Institutional Ethics Review Board of each center. Written informed consent was waived given the retrospective and anonymized nature of the data.

### Variable Definitions and Standard Echocardiography

A family history of SCD was defined as having at least one first-degree relative who experienced SCD before age 40 years or SCD occurring in a first-degree relative with confirmed HCM at any age. Nonsustained ventricular tachycardia (VT) was defined as at least 3 consecutive ventricular beats at a rate of ≥120 beats/min lasting <30 seconds. The 5-year SCD risk score was calculated based on the 2014 European Society of Cardiology guideline.^4^

Standard transthoracic echocardiography was performed for all patients. LV dimensions were measured at the standard parasternal views at end-diastole and end-systole. Maximal LV wall thickness was measured as the thickest myocardial segment at end-diastole. Left atrial (LA) volume was calculated by using the area-length method and indexed to body surface area. Mitral inflow velocities were measured at the tip of the mitral leaflets in the apical 4-chamber view. Tissue Doppler velocities were measured at the medial mitral valve annulus. Maximal LV outflow tract gradient was recorded at rest or with the Valsalva maneuver. LV apical aneurysm was defined by dyskinetic or akinetic LV apical segments with wall thinning, as observed on echocardiography or CMR imaging.

### LV Global Longitudinal Strain

LV-GLS was measured at the central core laboratory of Seoul National University Hospital by 2 experienced sonographers who were blinded to patient information. Two-dimensional echocardiographic DICOM (Digital Imaging and Communications in Medicine) images of the patients who were enrolled at Samsung Medical Center were transferred to Seoul National University Hospital via an external hard drive and analyzed using vendor-independent postprocessing software (Imaging Arena 4.6; TomTec Imaging Systems). Speckle-tracking analysis was performed by semi-automatically tracing the LV endocardial borders on 2-, 3-, and 4-chamber views at end-systole, with manual adjustments as needed. Myocardial motion was then tracked throughout the cardiac cycle, and LV-GLS was automatically calculated as the average of all segmental longitudinal strain values. For patients with atrial fibrillation, LV-GLS measurements were averaged over 3 cardiac cycles. The absolute value of LV-GLS was reported for straightforward interpretation. Excellent reproducibility of LV-GLS measurements in our laboratory has been previously reported, with an intraclass correlation coefficient of 0.96.^24^

### CMR Imaging

All patients underwent CMR imaging at the time of HCM diagnosis. The median interval between echocardiography and CMR was 28 days. Details regarding CMR scanners, field strengths, contrast agent dose, and administration timing are provided in Supplemental Table 1. At both centers, steady-state free precession cine images were obtained during breath-holding, and short-axis images were acquired at 10-mm intervals (6-mm slice thickness and 4-mm interslice gap) using retrospective electrocardiographic gating. Standard long-axis views covering the entire left ventricle were also acquired. LGE imaging in the long-axis, short-axis, and 4-chamber views was performed by using a phase-sensitive inversion recovery sequence 10 to 15 minutes after contrast administration. The inversion time was individually adjusted to null normal myocardium.

CMR image quantification was performed in a core laboratory located at Seoul National University Hospital using cvi42 software (Circle Cardiovascular Imaging). The DICOM CMR image files of patients from Samsung Medical Center were archived on a secure external hard drive and transferred to the core laboratory. Two independent observers (one experienced cardiologist [S.K.] and one cardiovascular imaging technician, each with >5 years of CMR experience), blinded to patients’ information and outcomes, analyzed the CMR images. Covering the entire left ventricle, endocardial and epicardial borders were automatically traced on all short-axis stacks at end-systole and end-diastole. The observers manually adjusted these borders to fit the anatomical contours. Papillary muscles were excluded from the cavity volume but included in the myocardial mass when calculating LV end-diastolic volume, end-systolic volume, and mass index, in accordance with the guideline recommendations.^25^

LGE was defined as areas with signal intensity ≥6 SDs above that of remote normal myocardium. The presence and extent of LGE were delineated semi-automatically in all short-axis slices from the LV base to apex and expressed as a percentage of total LV myocardial mass (LGE%). Interobserver variability was assessed between the observers in 15 randomly selected samples, demonstrating acceptable reproducibility (intraclass correlation coefficient = 0.81).

### Outcome Assessment

The primary outcome was defined as a composite of adverse CV events related to HCM, including CV death (which includes SCD), aborted SCD, appropriate ICD shock for terminating fatal ventricular arrhythmia, and admission for heart failure.^2^ CV death was defined as SCD or death attributed to myocardial infarction, progressive heart failure, major vascular disease, and cerebrovascular events. Mortality data were ascertained based on official national death records provided by Statistics Korea and official electronic medical records. In addition, SCD was further adjudicated through direct reports from family members, as well as information from emergency medical personnel and attending physicians, when applicable. Heart failure hospitalization was defined as admission requiring intravenous diuretics or inotropes due to worsening heart failure symptoms or pulmonary congestion. In addition to the primary outcome, an exploratory analysis was conducted for a secondary outcome comprising SCD-related events, defined as SCD, aborted SCD, or appropriate ICD shock.

### Statistical Analysis

Continuous variables are expressed as median (Q1-Q3), and categorical variables are expressed as frequencies with percentages. Group comparisons were performed by using the Kruskal-Wallis test for continuous variables and the chi-square test for categorical variables. Complete data were available for both LGE% and LV-GLS. The association between LGE% and LV-GLS was assessed by using Pearson correlation analysis. The correlation coefficient, 95% CI, and *P* value are reported. To visualize this relationship, a scatterplot was generated with a fitted linear regression line and a corresponding 95% confidence band. Patients were categorized based on median values of LGE% (≤4.2% vs >4.2%) and LV-GLS (≥14.3% vs <14.3%) across the entire cohort. In addition, alternative cutoff values derived from the maximally selected rank statistics (2.8% for LGE% and 9.7% for LV-GLS) were tested (Supplemental Figure 1). For the evaluation of SCD-related events, an additional analysis was performed by using the LGE% cutoff of ≥15% as proposed in the clinical guidelines.^3^ Kaplan-Meier curves were generated with 95% CI, and group comparisons were performed by using the log-rank test. In addition, prognosis was compared across 4 groups stratified according to LGE and LV-GLS.

Cox proportional hazards regression with a shared frailty term for the center was used to evaluate the association between risk factors and CV events, expressed as HRs with 95% CIs. Variables included in the multivariable model were selected based on their clinical importance and relevance to guideline-based risk stratification in HCM. Subgroup analyses were performed according to sex, age group (≤60 years vs >60 years), and HCM morphology (apical vs non-apical). Interactions between each subgroup variable and the LV-GLS and LGE% categories were tested in the Cox regression models by including interaction terms. The incremental prognostic value of LGE% and LV-GLS beyond the 5-year ESC SCD risk score was assessed by using the net reclassification improvement and integrated discrimination improvement indices.

To determine the potential mediating role of LV-GLS in the relationship between LGE and CV events, a structural equation model was constructed with 5-year CV events as the outcome. A single-mediator, parsimonious model was constructed with 3 core variables, estimating both the direct effect (LGE → 5-year CV event) and the indirect effect (LGE → LVGLS → 5-year CV event). This was based on our hypothesis that myocardial fibrosis (LGE) influences clinical outcomes both directly and indirectly via impaired LV systolic function. Standardized beta-coefficients, along with 95% CIs and corresponding *P* values, are reported. For the continuous-variable model, mediation percentages for the direct and indirect pathways were calculated as the proportion of each effect relative to the total effect (direct + indirect). Further details are provided in the Supplemental Methods.

All statistical analyses were performed by using R version 4.3.0 (R Foundation for Statistical Computing), and statistical significance was set at *P* < 0.05.

## Results

### Baseline Cohort Characteristics

A total of 652 patients with HCM were analyzed, with a median age of 56 years and male predominance (481 of 652 patients [73.8%]) (Table 1). Family history of SCD, nonsustained VT, and history of syncope were reported in 13.8% (90 of 652), 20.3% (118 of 582), and 12.3% (80 of 652) of patients, respectively. The median 5-year SCD risk score was 2.0% (Q1-Q3: 1.5%-3.2%).

**Table 1:** Baseline Characteristics of the Study Population (N = 652)

Age, y	56 (49-65)
Male	481 (73.8)
Body mass index, kg/m^2^	25.1 (23.3-27.0)
SBP, mm Hg	127 (115-138)
DBP, mm Hg	76 (68-82)
Heart rate, beats/min	69 (62-77)
NYHA functional class ≥Ⅲ	29 (4.4)
Family history of HCM	71 (10.9)
Family history of SCD	90 (13.8)
Nonsustained VT^a^	118 (20.3)
Syncope	80 (12.3)
5-year SCD risk score, %	2.0 (1.5-3.2)
Comorbidities	
Hypertension	253 (38.8)
Diabetes mellitus	96 (14.7)
Dyslipidemia	141 (21.6)
Atrial fibrillation	94 (14.4)
Previous PCI	4 (0.6)
Previous myocardial infarction	4 (0.6)
History of cancer	56 (8.6)
Echocardiography	
LV end-diastolic diameter, mm	48.0 (44.0-51.0)
LV end-systolic diameter, mm	28.0 (25.0-31.0)
LV ejection fraction, %	65.0 (60.0-69.0)
Maximal wall thickness, mm	17.9 (16.0-20.0)
Maximal wall thickness ≥30 mm	20 (3.1)
LA dimension, mm	43.5 (39.0-49.0)
LA volume index, mL/m^2^	41.3 (32.4-52.7)
E/A ratio	0.9 (0.7-1.3)
Septal eʹ-wave, cm/s	5.0 (4.0-6.0)
E/eʹ ratio	12.1 (9.7-15.8)
TR peak velocity, m/s	2.3 (2.2-2.5)
Maximal LVOT gradient, mm Hg	5.3 (3.7-13.3)
Maximal LVOT gradient ≥30 mm Hg^b^	121 (18.6)
LV-GLS, %	14.3 (11.4-17.5)
CMR	
LV end-diastolic volume, mL	140.8 (123.3-159.4)
LV end-systolic volume, mL	44.5 (36.0-54.3)
LV stroke volume, mL	94.3 (80.3-108.9)
LV ejection fraction by CMR, %	68.7 (63.6-72.3)
Cardiac output, L/min	6.1 (5.2-7.2)
LV mass index, g/m^2^	91.7 (76.5-113.1)
LGE%	4.2 (1.0-10.7)
LGE% ≥15%	107 (16.4)
Morphologic characteristics	
Apical HCM	161 (24.7)
LV apical aneurysm	67 (10.3)

Values are median (Q1-Q3) or n (%).

CMR = cardiovascular magnetic resonance; DBP = diastolic blood pressure; E/A ratio = ratio of the early (E) to late (A) ventricular filling velocities; E/eʹ = ratio between early mitral inflow velocity and mitral annular early diastolic velocity; HCM = hypertrophic cardiomyopathy; LA = left atrial; LGE = late gadolinium enhancement; LV = left ventricular; LVOT = left ventricular outflow tract; LV-GLS = left ventricular global longitudinal strain; PCI = percutaneous coronary intervention; SBP = systolic blood pressure; SCD = sudden cardiac death; TR = tricuspid regurgitation.

a Nonsustained ventricular tachycardia (VT) data were available in 89% of patients.

b Maximum value measured either at rest or during the Valsalva maneuver.

**Figure 1:**
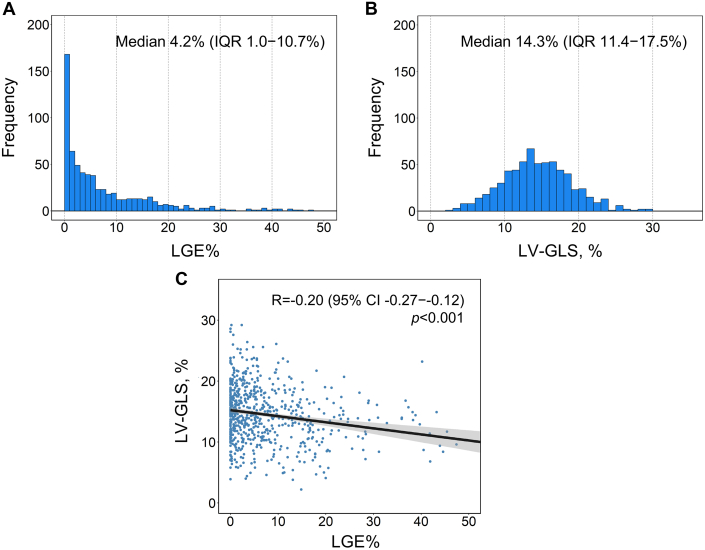
Distribution of LGE% and LV-GLS Measurements Across the Study Population (A) Histogram of late gadolinium enhancement (LGE%). (B) Histogram of left ventricular global longitudinal strain (LV-GLS). (C) Correlation plot between LGE% and LG-VLS.

Regarding echocardiographic parameters, the median maximal LV wall thickness was 17.9 mm (Q1-Q3: 16.0-20.0 mm). Measurement of LV ejection fraction yielded median values of 65.0% and 68.7% on echocardiography and CMR, respectively. Among them, 121 patients (18.6% [121 of 652]) exhibited an LV outflow tract gradient ≥30 mm Hg at rest or with the Valsalva maneuver. Figure 1 illustrates the distribution of LGE% and LV-GLS across the study population. The median LGE% was 4.2% (Q1-Q3: 1.0%-10.7%), with 107 patients (16.4% [107 of 652]) having measurements ≥15%. The median LV-GLS was 14.3% (Q1-Q3: 11.4%-17.5%). A weak inverse correlation was observed between LGE% and LV-GLS (Pearson’s *R* = –0.20; 95% CI: –0.27 to –0.12; *P* < 0.001).

Differences between centers are shown in Supplemental Table 2. Compared with patients from Samsung Medical Center, those from Seoul National University Hospital were older and had a higher 5-year SCD risk score and a lower LV ejection fraction.

### CV Events According to LGE and LV-GLS

During the median follow-up of 7.4 years (Q1-Q3: 3.4-10.3 years), 59 patients (9.0% [59 of 652]) experienced CV events, including 23 CV deaths (with 10 SCDs), 5 appropriate ICD shocks, and 38 admissions for heart failure. Univariable Cox analysis identified age, female sex, advanced symptoms, nonsustained VT, syncope, 5-year SCD risk score, atrial fibrillation, LV ejection fraction, LA size and volume index, *ratio* between early mitral inflow velocity and mitral annular early diastolic velocity, tricuspid regurgitation peak velocity, LV end-systolic volume, LV stroke volume, cardiac output, LV mass index, LV apical aneurysm, LV-GLS, and LGE% as significant predictors of CV events (Supplemental Table 3).

**Figure 2:**
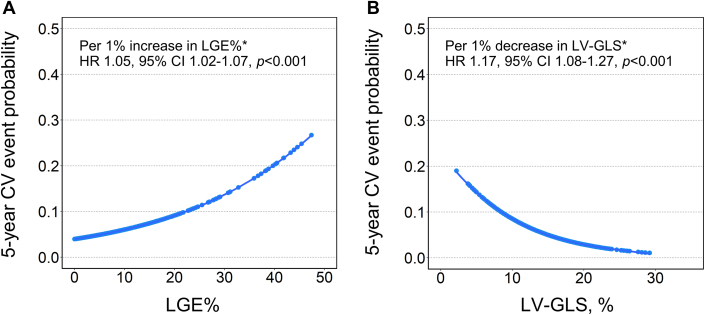
Relationship of LGE% and LV-GLS With 5-Year CV Event Probability (A) Relationship of LGE% with 5-year cardiovascular (CV) event probability. (B) Relationship of LV-GLS with 5-year CV event probability. ∗HRs for 5-year CV event are presented. Abbreviations as in Figure 1.

**Figure 3:**
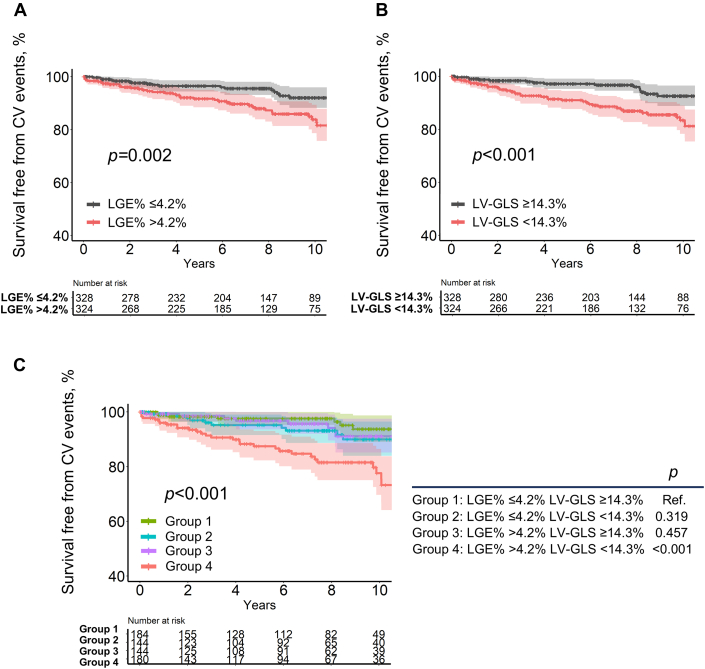
Cumulative CV Events According to LGE% and LV-GLS Survival free from CV events according to median LGE% (>4.2% vs ≤4.2%) (A), median LV-GLS (<14.3% vs ≥14.3%).(B), and median LGE% and LV-GLS (C). The shaded area represents the 95% CI. Ref. = reference; other abbreviations as in Figures 1 and 2.

A gradual increase in 5-year CV event probability was observed with increasing LGE% and decreasing LV-GLS (Figure 2). Multivariable Cox analysis revealed significant associations between increased LGE% and CV events (per 1% increase in LGE%, adjusted HR: 1.04; 95% CI: 1.01-1.06; *P* = 0.005), as well as between decreased LV-GLS and CV events (per 1% decrease in LV-GLS, adjusted HR: 1.08; 95% CI: 1.00-1.15; *P* = 0.038) (Table 2). When patients were categorized into 2 groups based on median LGE% (4.2%) and LV-GLS (14.3%), patients with an LGE% >4.2% and LV-GLS <14.3% exhibited significantly lower cumulative survival free from CV events compared with their counterparts (*P* = 0.002 and *P* < 0.001, respectively) (Figures 3A and 3B). Furthermore, LGE >4.2% (adjusted HR: 1.73; 95% CI: 0.95-3.15; *P* = 0.071) and LV-GLS <14.3% (adjusted HR: 1.94; 95% CI: 1.04-3.59; *P* = 0.036) were found to be associated with a higher risk of CV events. Similar findings were observed when using the cutoff values derived from the maximally selected rank statistics (LGE% >2.8%, adjusted HR: 3.44 [95% CI: 1.65-7.19; *P* = 0.001]; LV-GLS <9.7%, adjusted HR: 2.27 [95% CI: 1.23-4.19; *P* = 0.009]) (Supplemental Table 4).

**Table 2:** Association of LGE and LV-GLS With Cardiovascular Events

	Univariable Analysis		Multivariable Analysis^a^	
	HR (95% CI)	*P* Value	HR (95% CI)	*P* Value
LGE				
LGE% (per 1% increase)	1.04 (1.02-1.07)	<0.001	1.04 (1.01-1.06)	0.005
LGE% >4.2% vs ≤4.2%	2.38 (1.37-4.16)	0.002	1.73 (0.95-3.15)	0.071
LV-GLS				
LV-GLS, % (per 1% decrease)	1.12 (1.05-1.19)	<0.001	1.08 (1.00-1.15)	0.038
LV-GLS <14.3% vs ≥14.3%	2.67 (1.51-4.71)	<0.001	1.94 (1.04-3.59)	0.036
Groups by LGE and LV-GLS				
Group 1: LGE ≤4.2%, LV-GLS ≥14.3%	1 (Reference)		1 (Reference)	
Group 2: LGE ≤4.2%, LV-GLS <14.3%	1.59 (0.63-4.03)	0.329	1.25 (0.48-3.23)	0.643
Group 3: LGE >4.2%, LV-GLS ≥14.3%	1.36 (0.52-3.53)	0.531	1.09 (0.41-2.89)	0.862
Group 4: LGE >4.2%, LV-GLS <14.3%	4.57 (2.10-9.97)	<0.001	2.88 (1.21-6.85)	0.017

Abbreviations as in Table 1.

a Adjusted for age, sex, 5-year SCD risk score, atrial fibrillation, LV ejection fraction, and LV apical aneurysm.

### CV Events According to Both LGE% and LV-GLS

Further stratification of patients into 4 groups based on median LGE% and LV-GLS showed that patients with both LGE% >4.2% and LV-GLS <14.3% exhibited the highest SCD risk score, the highest prevalence of atrial fibrillation, and the lowest LV ejection fraction (Supplemental Table 5). The cumulative rate of CV events was the highest in this group, whereas the remaining 3 groups showed comparable survival rates (*P* < 0.001) (Figure 3C). Multivariable Cox analysis revealed a significantly higher risk of CV events in patients with both LGE% >4.2% and LV-GLS <14.3% compared with those with LGE% ≤4.2% and LV-GLS ≥14.3% (adjusted HR: 2.88; 95% CI: 1.21-6.85; *P* = 0.017) (Table 2). Similar findings were observed in the sensitivity analysis, which included only patients with preserved LV ejection fraction (≥50%) (n = 630) (Supplemental Figure 2). When using the cutoff values derived from the maximally selected rank statistics, patients with both LGE% >2.8% and LV-GLS <9.7% had a significantly higher risk of CV events compared with those with LGE% ≤2.8% and LV-GLS ≥9.7% (adjusted HR: 9.52; 95% CI: 3.19-28.35; *P* < 0.001) (Supplemental Table 4).

### Mediation Analysis Between LGE, LV-GLS, and CV Events

**Figure 4:**
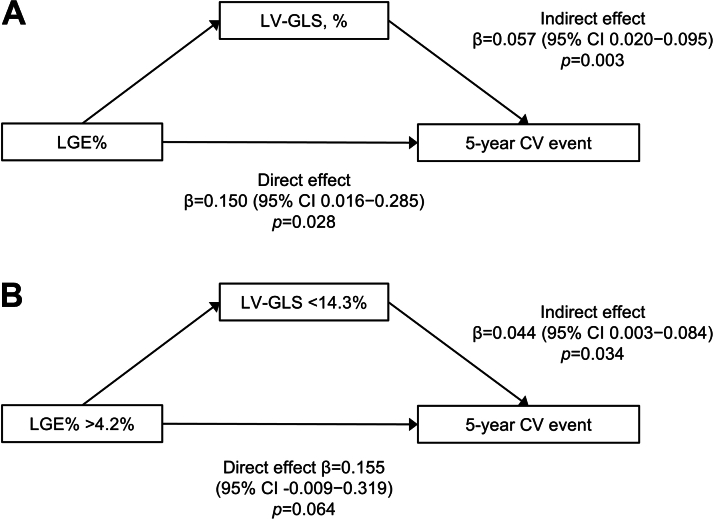
Mediation Analysis of LGE%, LV-GLS, and 5-Year CV Event A structural equation model shows the direct effect of LGE% on 5-year CV events and the indirect effect mediated by LV-GLS. (A) Model using LGE% and LV-GLS as continuous variables. (B) Model using LGE% and LV-GLS as categorical variables. Standardized beta-coefficients (β), along with 95% CIs and *P* values, are presented. Abbreviations as in Figures 1 and 2.

Mediation analysis showed a significant direct prognostic effect of LGE% on 5-year CV events (β = 0.150; 95% CI: 0.016-0.285; *P* = 0.028; mediation percentage 72.5%) and a significant indirect effect of LGE% mediated by LV-GLS (β = 0.057; 95% CI: 0.020-0.095; *P* = 0.003; mediation percentage 27.5%), resulting in a significant total effect (β = 0.207; 95% CI: 0.077-0.339; *P* = 0.002) (Figure 4A). Similar findings were observed when analyzing LGE% and LV-GLS categories, showing the significant indirect effect of LGE% >4.2% on 5-year CV events mediated by LV-GLS <14.3% (β = 0.044; 95% CI: 0.003-0.084; *P* = 0.034) (Figure 4B).

### SCD-Related Events

**Figure 5:**
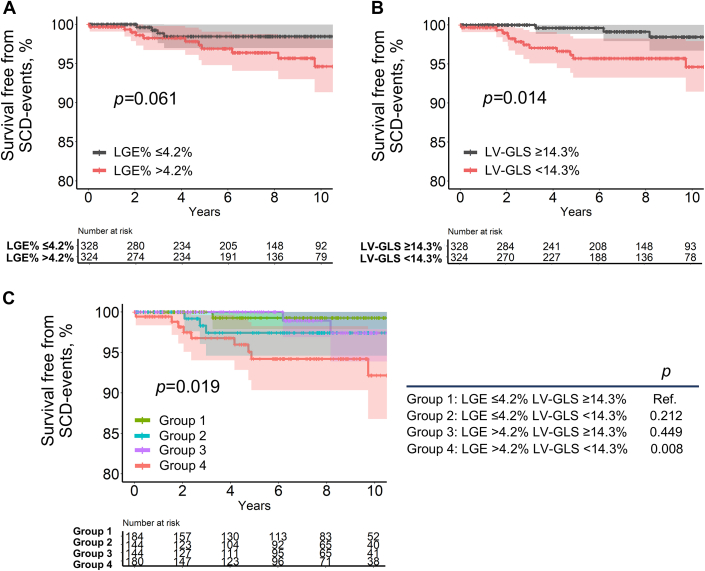
Cumulative SCD-Related Events According to LGE% and LV-GLS Survival free from sudden cardiac death (SCD)-related events according to median LGE% (>4.2% vs ≤4.2%) (A), median LV-GLS (<14.3% vs ≥14.3%) (B), and median LGE% and LV-GLS (C). The shaded area represents the 95% CI. Other abbreviations as in Figures 1 and 2.

During follow-up, 15 patients (2.3% [15 of 652]) experienced SCD-related events. Patients with a higher LGE% (>4.2%) exhibited a trend toward higher SCD-related events (3.4% [11 of 324]) compared with those with a lower LGE% (≤4.2%) (1.2% [4 of 328]) (*P* = 0.061) (Figure 5A). Moreover, patients with a lower LV-GLS (<14.3%) showed a significantly higher cumulative rate of SCD-related events compared with those with a higher LV-GLS (≥14.3%) (3.7% [12 of 324] vs 0.9% [3 of 328]; *P* = 0.014) (Figure 5B). Notably, SCD-related events most frequently occurred in patients with both high LGE% (>4.2%) and low LV-GLS (<14.3%) (9 of 15 SCD-related event cases [5.0% (9 of 180)]) (Figure 5C). Similar findings were observed when using the 15% cutoff for extensive LGE (Supplemental Figure 3), as well as using the LGE% and LV-GLS cutoffs identified by the maximally selected rank statistics (Supplemental Figure 4). The addition of LGE% and LV-GLS to the 5-year SCD risk score significantly improved risk stratification for 5-year SCD-related events, with a net reclassification improvement of 0.96 (95% CI: 0.53-1.39; *P* < 0.001) and an integrated discrimination improvement of 0.03 (95% CI: 0.01-0.06; *P* = 0.016).

#### Subgroup Analyses

Subgroup analyses showed that patients with both increased LGE and impaired LV-GLS had a higher risk of CV events compared with those with low LGE and preserved LV-GLS in both male (adjusted HR: 4.29; 95% CI: 1.24-14.85; *P* = 0.021) and female (adjusted HR: 3.29; 95% CI: 1.00-10.77; *P* = 0.049) patients, with no significant interaction by sex (*P*_interaction_ = 0.149) (Supplemental Table 6). There was no significant difference in the association of LGE and LV-GLS categories with CV events across age subgroups or HCM morphologic types.

## Discussion

This study made several key findings, summarized as follows. First, increased LGE% and decreased LV-GLS were each associated with a higher risk of adverse CV events in patients with HCM. Second, the risk of CV events was particularly higher among those with both higher LGE% and lower LV-GLS. Third, the prognostic impact of LGE on CV events was significantly mediated by LV systolic dysfunction, as assessed by LV-GLS, highlighting the role of LV systolic dysfunction in this relationship. Lastly, patients with both higher LGE% and lower LV-GLS were also at a higher risk for SCD-related events. Collectively, these results underscore the importance of integrating both imaging markers into the risk stratification of patients with HCM (Central Illustration).

**Central Illustration:**
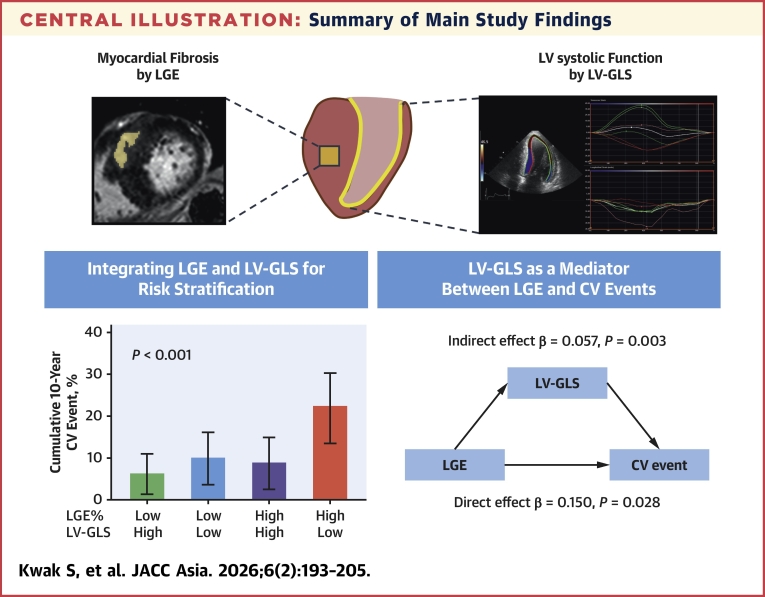
Summary of Main Study Findings (Upper panel) Myocardial fibrosis can be quantified by using cardiovascular magnetic resonance as a structural marker of left ventricular (LV) remodeling, while LV systolic dysfunction can be assessed with left ventricular global longitudinal strain (LV-GLS). Together, these assessments provide a comprehensive phenotyping of LV remodeling in hypertrophic cardiomyopathy (HCM). (Lower left panel) When patients were stratified into 4 groups based on the median values of late gadolinium enhancement (LGE%) and LV-GLS, the highest incidence of cardiovascular (CV) events was observed in those with both high LGE and low LV-GLS, whereas the other 3 groups had similar incidence rates. Low LGE indicates LGE ≤4.2%, and high LGE indicates >4.2%. Low LV-GLS indicates <14.3%, and high LV-GLS indicates ≥14.3%. (Lower right panel) In the mediation analysis, there was a significant direct effect of LGE% on CV events, as well as a significant indirect effect of LGE% mediated through LV-GLS.

Although advancements in HCM management have significantly reduced SCD incidence,^26^ it is still regarded as one of the most devastating complications. Appropriate primary prevention for individuals with HCM continues to be clinically challenging,^3^^,^^4^ and the risk of adverse CV events, including progressive heart failure, remains substantially high.^2^ Therefore, identifying novel biomarkers to refine risk stratification and better delineate risk factor patterns is essential for optimizing both surveillance and prevention strategies.^27^

LGE serves as a marker for myocardial fibrosis in patients with HCM, observed in any hypertrophied segment of the left ventricle, not aligning with coronary artery territory.^19^ LGE is observed in 50% to 70% of HCM cases^6^ and is linked to increased ventricular tachyarrhythmia incidence and inducibility.^28^ Over the disease course, LGE tends to increase in parallel with worsening systolic function,^16^ ultimately contributing to end-stage HCM and adverse outcomes.^14^ Although 15% is the recommended LGE% cutoff for extensive LGE requiring an ICD for primary prevention, emerging evidence suggests that arrhythmic SCD risk may increase gradually even at lower LGE% thresholds.^7^^,^^8^^,^^10^ Deep phenotyping of LGE, including radiomic analysis^29^ or scar characterization,^30^ may offer additional prognostic value for stratifying SCD risk. Despite the small number of SCD-related events in the current study, LGE% was able to effectively discriminate SCD risk, regardless of the cutoff applied. As such, our findings support the use of LGE in risk stratification of SCD-related events in patients with HCM.

LV systolic function is an important determinant of prognosis in HCM, including the risk of heart failure and SCD. Studies have shown that LV-GLS is a sensitive marker of subclinical LV systolic dysfunction, and its prognostic value has been well established in patients with HCM.^22^ In addition to the poor outcomes associated with end-stage HCM,^31^ a low-normal LV ejection fraction (50%-60%) has also been linked to adverse outcomes compared with preserved LV ejection fraction (>60%).^32^ Even within the low-normal range, LV-GLS can further stratify risk. A recent study analyzing 349 patients with HCM and low-normal LV ejection fraction (50%-60%) found that LV-GLS was independently associated with a composite of CV death and SCD-related events.^23^ Therefore, incorporating LV-GLS into risk stratification may offer incremental prognostic value. Indeed, the combined use of LV-GLS and LA reservoir strain has been shown to provide significant predictive value for SCD.^21^ Therefore, the integrated use of multiple imaging biomarkers may ultimately help redefine disease staging in HCM.

Mediation analysis confirmed the significant mediating effect of LV-GLS in the relationship between LGE and CV events. These findings are consistent with outcomes observed across 4 groups stratified by LGE and LV-GLS: patients with high LGE but preserved LV-GLS were not significantly associated with increased CV risk, whereas those with both high LGE and impaired LV-GLS experienced the highest event rates. These results suggest a stepwise mechanism in which myocardial fibrosis, detected by LGE, reflects adverse structural remodeling of the left ventricle, which subsequently leads to systolic dysfunction and worse outcomes. The prognostic effect of fibrosis seems to be amplified when accompanied by functional deterioration, as captured by impaired LV-GLS. This hypothesis warrants further validation through longitudinal follow-up using both CMR and echocardiographic imaging. Although strain imaging may not be routinely available for all patients with HCM, our findings suggest that it should be considered in those with borderline or substantial LGE, as it may offer incremental prognostic information beyond the extent of LGE alone.

LGE% can vary substantially among patients with preserved LV ejection fraction.^8^ In the current study, LGE% and LV-GLS showed only a weak correlation (Pearson’s *R* = –0.20), suggesting that these variables capture distinct characteristics of the left ventricle: myocardial fibrosis, as detected by LGE, and systolic dysfunction, as detected by LV-GLS. Accordingly, LGE and LV-GLS seem to provide independent and complementary prognostic information, with worse outcomes observed in patients with both increased LGE and impaired LV-GLS. Despite the male predominance in the current cohort, this finding was consistently observed when analyzed separately in men and women, suggesting that the result may be generalizable to both sexes. In addition, given the suggested association of LGE and LV systolic dysfunction with SCD risk, assessing both markers could improve the identification of high-risk individuals for ICD implantation in the primary prevention setting. With ongoing advancements in the therapeutic landscape of HCM, such as cardiac myosin inhibitors,^33^ the integration of LGE and LV-GLS into the risk stratification process may facilitate the development of tailored strategies in HCM management.

### Study Limitations

First, although we gathered a substantial number of participants with both CMR and echocardiographic strain imaging, this study was conducted retrospectively. Therefore, potential selection bias and unmeasured confounders may be present. Second, the relatively small number of SCD-related events limited our ability to rigorously assess the independent prognostic value of LGE and LV-GLS for SCD prediction and to determine optimal cutoff values. This may be partly because patients with an ICD at baseline or a history of aborted SCD (ie, those at high risk for SCD) were excluded. Nevertheless, the high crude rate of SCD-related events in patients with both high LGE and low LV-GLS suggests that the combined use of these imaging markers may enable more accurate risk prediction. Further validation in a larger cohort with more SCD-related events is needed to generalize our findings. Third, more detailed clinical information, including genetic testing, was unavailable in this study. Fourth, although the CMR quantification was performed in a core laboratory, differences in CMR scanners and magnetic field strengths between study centers may have introduced variability in the measurements (Supplemental Table 1). In addition, extracellular volume fraction using T1-mapping analysis could provide further insights into the prognostic role of diffuse interstitial myocardial fibrosis, warranting future studies. Fifth, longitudinal assessments of LGE and LV-GLS, which were not conducted in the current study, could provide further insights into the natural course and impact of myocardial fibrosis on systolic dysfunction. Lastly, our mediation analysis was exploratory and hypothesis-generating, based on a parsimonious and simplified model. Although this approach provides mechanistic insight into the effects of LGE via LV systolic dysfunction, further studies incorporating more comprehensive covariate adjustment and external validation are warranted.

## Conclusions

LGE and LV-GLS provided complementary prognostic information in HCM. Patients with both increased LGE and decreased LV-GLS were identified as a high-risk group with a particularly poor prognosis. The combined use of these imaging biomarkers is expected to improve risk stratification in HCM.

### Data Availability

The data supporting the findings of this study will be available from the corresponding authors upon reasonable request.

## Funding Support and Author Disclosures

This study was supported by a research grant from the 2021 Seoul National University Research Fund (800-20210548). The authors have reported that they have no relationships relevant to the contents of this paper to disclose.
