# Supplementary material for: Integrated Use of Late Gadolinium Enhancement and Left Ventricular Global Longitudinal Strain in Hypertrophic Cardiomyopathy
Source: JACC Asia. 2026 Feb 3;6(2):193–205. doi: 10.1016/j.jacasi.2025.07.026 (PMC12904828; doi:10.1016/j.jacasi.2025.07.026)
Supplement: Supplementary Material [file mmc1.docx]

**Supplemental Methods.**

**Development of structural equation model**

To evaluate the potential mediating role of LV-GLS in the relationship between LGE and 5-year CV events, we constructed a single-mediator structural equation model (SEM) involving three core variables: LGE, LV-GLS, and CV outcome. The structure was pre-specified based on a clinically grounded hypothesis that myocardial fibrosis contributes to adverse outcomes both directly and indirectly via impaired LV systolic function.

Two separate models were evaluated: one using continuous forms of LGE and LV-GLS, and another using dichotomized (median-split) categorical variables.


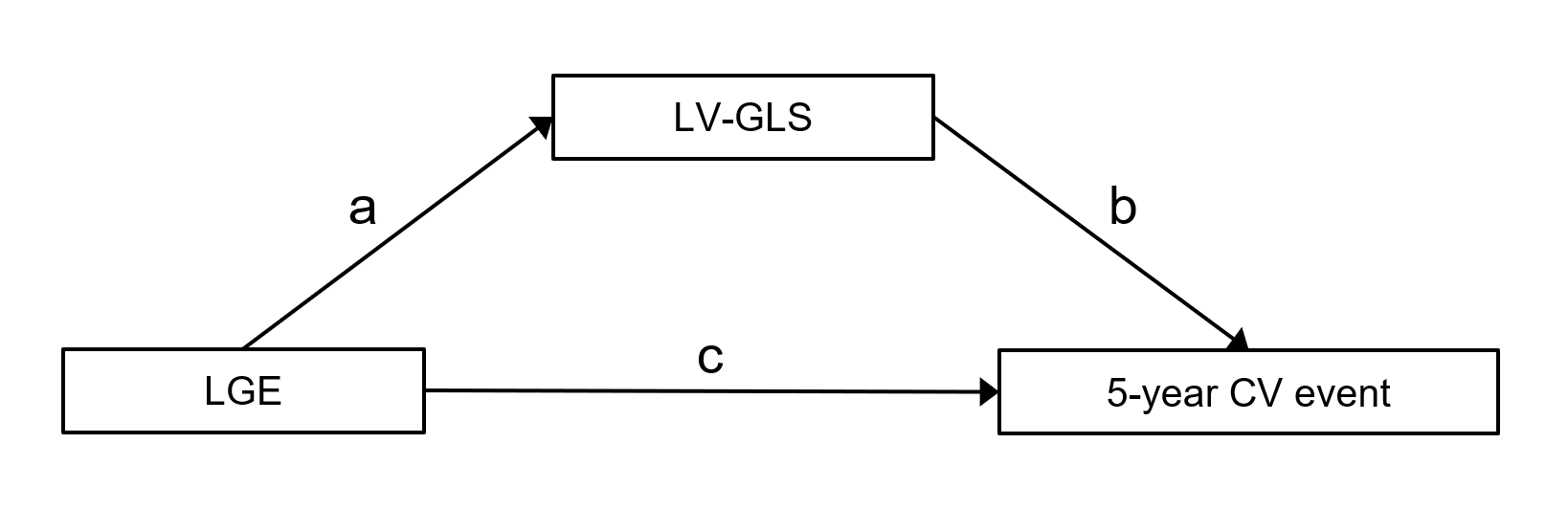


Path a: LGE → LV-GLS

Path b: LV-GLS → 5-year CV event

Path c: LGE → 5-year CV event (direct path)

Indirect effect: a × b

Total effect: c + (a × b)

In the continuous-variable model, the relationship from LGE% to LV-GLS was modeled using linear regression, while the paths from both LGE% and LVGLS to the binary outcome (5-year CV events) were modeled using probit regression. In the categorical-variable model, both LGE and LV-GLS were treated as ordered categorical variables, and effects were estimated using probit link functions with the Weighted Least Squares Mean and Variance Adjusted (WLSMV) estimator.

The SEM included direct (LGE → CV outcome) and indirect (LGE → LVGLS → CV outcome) pathways, with total effects computed as the sum of both. Standardized beta coefficients, 95% confidence intervals, and *p*-values were derived. The proportion mediated was calculated as the ratio of the indirect effect to the total effect for the continuous-variable model. Model fit indices indicated excellent structural fit for both models, with a Comparative Fit Index (CFI) of 1.000, Root Mean Square Error of Approximation (RMSEA) of 0.000, and Standardized Root Mean Square Residual (SRMR) of 0.000, supporting the adequacy of the model.

**Supplemental Table 1. Technical details of cardiovascular magnetic resonance by study centers.**

| **Site** | **Scanner** | **Contrast agent, dose and timing** | **N** |
| --- | --- | --- | --- |
| Samsung Medical Center | Siemens MAGNETOM Avanto 1.5T | Gadobutrol 0.10−0.15 mmol/kg, 10−15 mins | 347 |
| Seoul National University Hospital | Siemens MAGNETOM Sonata 1.5T | Magnevist 0.20 mmol/kg OR  Dotarem 0.20 mmol/kg, 10−15 mins | 305 |
|  | Siemens MAGNETOM Trio 3T |  |  |
|  | Siemens MAGNETOM Skyra 3T |  |  |

**Supplemental Table 2.** **Baseline characteristics of the study population by participating center.**

| **Variables** | **SMC**  **(n=347)** | **SNUH**  **(n=305)** | ***p*** |
| --- | --- | --- | --- |
| Age, years | 54 (47−62) | 58 (50−67) | <0.001 |
| Male | 275 (79.3) | 206 (67.5) | 0.001 |
| Body mass index, kg/m² | 25.2 (23.4−27.1) | 25.1 (23.1−27.0) | 0.315 |
| SBP, mmHg | 126 (114−139) | 128 (117−137) | 0.779 |
| DBP, mmHg | 77 (67−85) | 74 (69−80) | 0.047 |
| HR, bpm | 70 (63−78) | 67 (60−76) | 0.020 |
| NYHA ≥Ⅲ | 3 (0.9) | 26 (8.5) | <0.001 |
| Family history of HCM | 33 (9.5) | 38 (12.5) | 0.138 |
| Family history of SCD | 52 (15.0) | 38 (12.5) | 0.413 |
| Non-sustained VT^a^ | 42 (13.7) | 76 (27.6) | <0.001 |
| Syncope | 30 (8.6) | 50 (16.4) | 0.004 |
| 5-year SCD risk score, % | 1.8 (1.4−3.0) | 2.2 (1.5−3.4) | 0.001 |
| ***Comorbidities*** |  |  |  |
| Hypertension | 138 (39.8) | 115 (37.7) | 0.646 |
| Diabetes mellitus | 44 (12.7) | 52 (17.0) | 0.144 |
| Dyslipidemia | 63 (18.2) | 78 (25.6) | 0.028 |
| Atrial fibrillation | 51 (14.7) | 43 (14.1) | 0.916 |
| Previous PCI | 0 (0.0) | 4 (1.3) | 0.102 |
| Previous myocardial infarction | 2 (0.6) | 2 (0.7) | >0.99 |
| History of cancer | 31 (8.9) | 25 (8.2) | 0.845 |
| ***Echocardiography*** |  |  |  |
| LV end-diastolic diameter, mm | 48.0 (45.0−52.0) | 46.0 (43.0−51.0) | 0.001 |
| LV end-systolic diameter, mm | 28.0 (26.0−31.0) | 28.0 (25.0−31.0) | 0.303 |
| LV ejection fraction, % | 66.0 (61.0−70.0) | 64.0 (59.0−68.0) | 0.002 |
| Maximal wall thickness, mm | 17.0 (16.0−20.0) | 18.0 (16.0−20.8) | 0.018 |
| Maximal wall thickness ≥30 mm | 9 (2.6) | 11 (3.6) | 0.603 |
| LA dimension, mm | 43.0 (39.0−47.0) | 45.0 (41.0−50.5) | <0.001 |
| LA volume index, mL/m^2^ | 40.5 (32.0−50.5) | 42.1 (32.5−56.1) | 0.287 |
| E/A ratio | 1.0 (0.7−1.3) | 0.8 (0.7−1.2) | 0.019 |
| Septal eʹ-wave, cm/s | 5.3 (4.0−6.6) | 4.4 (3.5−5.4) | <0.001 |
| E/eʹ ratio | 11.5 (9.2−14.9) | 12.9 (10.0−17.6) | <0.001 |
| TR peak velocity, m/s | 2.3 (2.1−2.6) | 2.3 (2.2−2.5) | 0.311 |
| Maximal LV outflow tract gradient, mmHg | 5.3 (3.5−20.9) | 5.3 (3.8−10.6) | 0.898 |
| Maximal LVOT gradient ≥30 mmHg^b^ | 74 (21.3) | 47 (15.4) | 0.066 |
| LV-GLS, % | 13.8 (11.3−16.9) | 14.8 (11.7−18.0) | 0.023 |
| ***CMR*** |  |  |  |
| LV end-diastolic volume, mL | 141.9 (124.9−161.3) | 139.7 (119.3−158.2) | 0.074 |
| LV end-systolic volume, mL | 45.0 (36.7−54.0) | 44.2 (35.4−54.7) | 0.896 |
| LV stroke volume, mL | 97.2 (83.4−111.7) | 91.5 (78.8−105.4) | 0.001 |
| LV ejection fraction by CMR, % | 69.3 (64.6−72.6) | 68.2 (62.4−72.0) | 0.032 |
| Cardiac output, L/min | 6.4 (5.4−7.4) | 5.7 (4.9−6.8) | <0.001 |
| LV mass index, g/m^2^ | 88.0 (74.8−108.0) | 96.4 (79.0−118.6) | 0.001 |
| LGE% | 3.8 (0.5−11.2) | 4.3 (1.2−9.8) | 0.113 |
| LGE% ≥15% | 64 (18.4) | 43 (14.1) | 0.165 |
| **Morphological pattern** |  |  |  |
| Apical HCM | 81 (23.3) | 80 (26.2) | 0.446 |
| LV apical aneurysm, n (%) | 46 (13.3) | 21 (6.9) | 0.011 |

Continuous variables are expressed as the median (interquartile range), and categorical variables are expressed as frequency (percentage).

^a^Non-sustained VT data was available in 89% of patients.

^b^Maximum value measured either at rest or during the Valsalva maneuver

bpm, beats per minute; CMR, cardiovascular magnetic resonance; HCM, hypertrophic cardiomyopathy; HR, heart rate; LA, left atrial; LGE, late gadolinium enhancement; LV, left ventricular; LVOT, left ventricular outflow tract; LV-GLS, left ventricular global longitudinal strain; NYHA, New York Heart Association; PCI, percutaneous coronary intervention; S(D)BP, systolic (diastolic) blood pressure; SCD, sudden cardiac death; TR, tricuspid regurgitation; VT, ventricular tachycardia

**Supplemental Table 3. Univariable Cox analysis for CV events.**

|  | **Hazard ratio**  **(95% CI)** | ***p*** |
| --- | --- | --- |
| Age, years | 1.03 (1.00−1.05) | 0.028 |
| Male | 0.55 (0.32−0.94) | 0.028 |
| Body mass index, kg/m² | 1.00 (0.92−1.09) | 0.979 |
| HR, bpm | 1.01 (0.99−1.03) | 0.366 |
| NYHA ≥Ⅲ | 9.17 (4.60−18.30) | <0.001 |
| Family history of HCM | 1.53 (0.75−3.12) | 0.243 |
| Family history of SCD | 1.23 (0.62−2.44) | 0.548 |
| Non-sustained VT^a^ | 1.98 (1.08−3.63) | 0.028 |
| Syncope | 2.24 (1.16−4.32) | 0.017 |
| 5-year SCD risk score, % | 1.13 (1.07−1.20) | <0.001 |
| Hypertension | 0.83 (0.49−1.41) | 0.496 |
| Diabetes mellitus | 1.59 (0.86−2.94) | 0.143 |
| Dyslipidemia | 1.27 (0.70−2.28) | 0.431 |
| Atrial fibrillation | 5.57 (3.33−9.32) | <0.001 |
| Previous PCI | − | - |
| History of cancer | 1.24 (0.56−2.76) | 0.603 |
| ***Echocardiography*** |  |  |
| LV end-diastolic diameter, mm | 1.00 (0.95−1.04) | 0.889 |
| LV end-systolic diameter, mm | 1.05 (0.99−1.11) | 0.119 |
| LV ejection fraction, % | 0.94 (0.91−0.98) | <0.001 |
| Maximal wall thickness, mm | 1.03 (0.98−1.09) | 0.209 |
| Maximal wall thickness ≥30 mm | 0.79 (0.18−3.46) | 0.754 |
| LA dimension, mm | 1.13 (1.09−1.16) | <0.001 |
| LA volume index, mL/m^2^ | 1.03 (1.02−1.03) | <0.001 |
| E/A ratio | 1.36 (0.97−1.89) | 0.071 |
| Septal eʹ-wave, cm/s | 0.87 (0.74−1.02) | 0.086 |
| E/eʹ ratio | 1.06 (1.03−1.09) | <0.001 |
| TR peak velocity, m/s | 3.55 (1.70−7.40) | <0.001 |
| Maximal LVOT gradient ≥30 mmHg | 1.50 (0.83−2.71) | 0.177 |
| LV-GLS, per 1% decrease | 1.12 (1.05−1.19) | <0.001 |
| **CMR** |  |  |
| LV end-diastolic volume, per 10 mL increase | 0.95 (0.87−1.05) | 0.306 |
| LV end-systolic volume, per 10 mL increase | 1.23 (1.10−1.37) | <0.001 |
| LV stroke volume, mL | 0.97 (0.96−0.98) | <0.001 |
| LV ejection fraction by CMR, % | 0.93 (0.91−0.96) | <0.001 |
| Cardiac output, L/min | 0.80 (0.66−0.96) | 0.018 |
| LV mass index, per 10 g/m^2^ increase | 1.09 (1.01−1.17) | 0.034 |
| LGE% | 1.04 (1.02−1.07) | <0.001 |
| LGE% ≥15% | 2.08 (1.16−3.71) | 0.013 |
| LV apical aneurysm | 2.12 (1.10−4.08) | 0.025 |

^a^Non-sustained VT data was available in 89% of patients.

bpm, beats per minute; CV, cardiovascular; CMR, cardiovascular magnetic resonance; CI, confidence interval; HCM, hypertrophic cardiomyopathy; HR, heart rate; LA, left atrial; LGE, late gadolinium enhancement; LV, left ventricular; LVOT, left ventricular outflow tract; LV-GLS, left ventricular global longitudinal strain; NYHA, New York Heart Association; PCI, percutaneous coronary intervention; SCD, sudden cardiac death; TR, tricuspid regurgitation; VT, ventricular tachycardia

**Supplemental Table 4. Association of LGE and LV-GLS with adverse CV events using cutoff values derived from maximally selected rank statistics.**

|  | **Univariable analysis** | | | **Multivariable analysis^a^** | |
| --- | --- | --- | --- | --- | --- |
|  | **HR (95% CI)** | ***p*** | **HR (95% CI)** | | ***p*** |
| **LGE** |  |  |  | |  |
| LGE% ≤2.8% (273/652, 41.9%) | 1 (ref.) |  | 1 (ref.) | |  |
| LGE% >2.8% (379/652, 58.1%) | 4.34 (2.13−8.83) | <0.001 | 3.44 (1.65−7.19) | | 0.001 |
| **LV-GLS** |  |  |  | |  |
| LV-GLS ≥9.7% (560/652, 85.9%) | 1 (ref.) |  | 1 (ref.) | |  |
| LV-GLS <9.7% (92/652, 14.1%) | 3.03 (1.74−5.29) | <0.001 | 2.27 (1.23−4.19) | | 0.009 |
| **Groups by LGE and LV-GLS** |  |  |  | |  |
| Group 1: LGE ≤2.8%, LV-GLS ≥9.7% (242/652, 37.1%) | 1 (ref.) |  | 1 (ref.) | |  |
| Group 2: LGE ≤2.8%, LV-GLS <9.7% (31/652, 4.8%) | 6.41 (1.72−23.89) | 0.006 | 5.58 (1.46−21.35) | | 0.012 |
| Group 3: LGE >2.8%, LV-GLS ≥9.7% (318/652, 48.8%) | 5.78 (2.26−14.57) | <0.001 | 4.96 (1.90−12.92) | | 0.001 |
| Group 4: LGE >2.8%, LV-GLS <9.7% (61/652, 9.4%) | 13.88 (4.99−38.57) | <0.001 | 9.52 (3.19−28.35) | | <0.001 |

^a^Adjusted for age, sex, 5-year SCD risk score, atrial fibrillation, LV ejection fraction, and LV apical aneurysm.

CI, confidence interval; HR, hazard ratio; LGE, late gadolinium enhancement; LV, left ventricular; LV-GLS, left ventricular global longitudinal strain; NYHA, New York Heart Association; SCD, sudden cardiac death

**Supplemental Table 5. Baseline characteristics of the study population according to LGE and LV-GLS.**

|  | **Groups categorized by LGE% and LV-GLS** | | | |  |
| --- | --- | --- | --- | --- | --- |
| **Variables** | **LGE% ≤4.2%**  **LV-GLS ≥14.3%**  **(n=184)** | **LGE% ≤4.2%**  **LV-GLS <14.3%**  **(n=144)** | **LGE% >4.2%**  **LV-GLS ≥14.3%**  **(n=144)** | **LGE% >4.2%**  **LV-GLS <14.3%**  **(n=180)** | ***p*** |
| Age, years | 58 (48−67) | 58 (51−66) | 53 (46−62) | 56 (47−65) | 0.021 |
| Male | 117 (63.6) | 108 (75.0) | 105 (72.9) | 151 (83.9) | <0.001 |
| Body mass index, kg/m² | 24.8 (23.1−26.8) | 25.4 (23.3−27.6) | 24.6 (23.0−26.8) | 25.6 (23.7−27.2) | 0.052 |
| SBP, mmHg | 128 (117−140) | 130 (117−141) | 120 (110−133) | 128 (115−137) | 0.001 |
| DBP, mmHg | 75 (67−81) | 78 (70−85) | 73 (65−80) | 78 (70−84) | <0.001 |
| HR, bpm | 66 (61−74) | 72 (65−81) | 66 (60−76) | 72 (64−81) | <0.001 |
| NYHA ≥Ⅲ | 5 (2.7) | 8 (5.6) | 6 (4.2) | 10 (5.6) | 0.520 |
| Family history of HCM | 22 (12.0) | 5 (3.5) | 21 (14.6) | 23 (12.8) | 0.007 |
| Family history of SCD | 30 (16.3) | 11 (7.6) | 23 (16.0) | 26 (14.4) | 0.102 |
| Non-sustained VT^a^ | 16 (9.8) | 21 (16.7) | 30 (22.2) | 51 (32.3) | <0.001 |
| Syncope | 23 (12.5) | 12 (8.3) | 20 (13.9) | 25 (13.9) | 0.412 |
| 5-year SCD risk score, % | 1.7 (1.3−2.8) | 1.7 (1.3−2.5) | 2.1 (1.5−3.4) | 2.7 (1.8−4.1) | <0.001 |
| ***Comorbidities*** |  |  |  |  |  |
| Hypertension | 70 (38.0) | 70 (48.6) | 40 (27.8) | 73 (40.6) | 0.004 |
| Diabetes mellitus | 32 (17.4) | 25 (17.4) | 15 (10.4) | 24 (13.3) | 0.236 |
| Dyslipidemia | 34 (18.5) | 30 (20.8) | 32 (22.2) | 45 (25.0) | 0.500 |
| Atrial fibrillation | 9 (4.9) | 22 (15.3) | 21 (14.6) | 42 (23.3) | <0.001 |
| Previous PCI | 1 (0.5) | 0 (0.0) | 2 (1.4) | 1 (0.6) | 0.506 |
| Previous myocardial infarction | 0 (0.0) | 1 (0.7) | 2 (1.4) | 1 (0.6) | 0.461 |
| History of cancer | 16 (8.7) | 14 (9.7) | 10 (6.9) | 16 (8.9) | 0.860 |
| ***Echocardiography*** |  |  |  |  |  |
| LV end-diastolic diameter, mm | 47.5 (44.0−51.5) | 48.0 (45.0−51.0) | 46.0 (42.0−50.5) | 48.0 (44.0−51.0) | 0.040 |
| LV end-systolic diameter, mm | 28.0 (25.0−31.0) | 29.0 (26.0−31.0) | 27.0 (24.0−30.0) | 28.0 (26.0−32.0) | 0.009 |
| LV ejection fraction, % | 66.0 (61.5−70.0) | 64.0 (59.0−69.0) | 65.5 (60.0−69.0) | 64.0 (58.0−68.0) | 0.001 |
| Maximal wall thickness, mm | 17.0 (15.2−19.0) | 17.0 (16.0−20.0) | 18.0 (16.0−20.0) | 19.0 (17.0−22.0) | <0.001 |
| Maximal wall thickness ≥30 mm | 0 (0.0) | 2 (1.4) | 6 (4.2) | 12 (6.7) | 0.001 |
| LA dimension, mm | 42.0 (39.0−47.0) | 43.0 (39.0−47.9) | 43.1 (39.4−49.0) | 46.0 (42.0−51.0) | <0.001 |
| LA volume index, mL/m^2^ | 39.2 (30.8−49.3) | 39.0 (30.5−49.0) | 41.3 (34.0−53.9) | 45.0 (35.8−57.4) | <0.001 |
| E/A ratio | 0.9 (0.7−1.3) | 0.8 (0.7−1.2) | 1.0 (0.8−1.4) | 0.9 (0.7−1.2) | 0.031 |
| Septal eʹ-wave, cm/s | 5.2 (4.2−6.4) | 5.0 (3.8−6.0) | 5.0 (4.0−6.0) | 4.3 (3.4−5.7) | <0.001 |
| E/eʹ ratio | 10.9 (8.9−14.0) | 12.0 (9.3−15.2) | 12.7 (10.2−15.9) | 13.3 (10.0−18.0) | <0.001 |
| TR peak velocity, m/s | 2.4 (2.2−2.6) | 2.4 (2.2−2.5) | 2.4 (2.2−2.5) | 2.3 (2.2−2.6) | 0.721 |
| Maximal LV outflow tract gradient,  mmHg | 5.3 (4.0−15.3) | 5.1 (3.5−11.8) | 6.3 (4.0−11.1) | 4.7 (3.1−16.8) | 0.113 |
| Maximal LVOT gradient ≥30 mmHg | 38 (20.7) | 23 (16.0) | 27 (18.8) | 33 (18.3) | 0.758 |
| LV apical aneurysm, n (%) | 9 (4.9) | 14 (9.7) | 7 (4.9) | 37 (20.6) | <0.001 |
| LV-GLS, % | 17.6 (16.2−20.1) | 11.6 (9.7−13.1) | 17.1 (15.5−19.0) | 11.1 (8.9−13.0) | <0.001 |
| ***CMR*** |  |  |  |  |  |
| LV end-diastolic volume, mL | 138.5 (120.7−157.8) | 136.3 (119.3−154.9) | 146.4 (124.9−163.7) | 141.7 (125.2−165.3) | 0.023 |
| LV end-systolic volume, mL | 41.5 (35.1−50.2) | 41.9 (34.6−50.6) | 46.3 (36.0−55.9) | 48.0 (39.1−62.0) | <0.001 |
| LV stroke volume, mL | 95.0 (81.5−109.4) | 94.2 (79.3−107.6) | 96.0 (81.5−112.2) | 92.4 (78.5−106.6) | 0.173 |
| LV ejection fraction by CMR, % | 69.5 (66.4−72.9) | 69.7 (64.7−72.9) | 69.3 (63.7−72.5) | 65.5 (58.9−70.7) | <0.001 |
| Cardiac output, L/min | 6.0 (5.1−7.0) | 6.2 (5.3−7.3) | 6.0 (5.2−7.1) | 6.2 (5.3−7.2) | 0.687 |
| LV mass index, g/m^2^ | 78.5 (70.4−92.0) | 94.7 (81.1−113.4) | 88.3 (75.8−107.3) | 107.6 (91.2−137.7) | <0.001 |
| LGE% | 0.9 (0.0−2.1) | 1.1 (0.0−2.6) | 8.9 (5.5−13.8) | 13.1 (7.3−18.5) | <0.001 |
| LGE% ≥15% | 0 (0.0) | 0 (0.0) | 29 (20.1) | 78 (43.3) | <0.001 |

^a^Data available in 89% of patients.

bpm, beats per minute; CMR, cardiovascular magnetic resonance; HCM, hypertrophic cardiomyopathy; HR, heart rate; LA, left atrial; LGE, late gadolinium enhancement; LV, left ventricular; LVOT, left ventricular outflow tract; LV-GLS, left ventricular global longitudinal strain; NYHA, New York Heart Association; PCI, percutaneous coronary intervention; S(D)BP, systolic (diastolic) blood pressure; SCD, sudden cardiac death; TR, tricuspid regurgitation; VT, ventricular tachycardia

**Supplemental Table 6. Association of LGE and LV-GLS categories with adverse CV events across subgroups.**

| **Subgroup** | **Group** | **Adjusted HR^a^**  **(95% CI)** | ***p*** | ***p*-for-interaction** |
| --- | --- | --- | --- | --- |
| Sex: Male (n=481) | Group 1: LGE ≤4.2%, LV-GLS ≥14.3% | 1 (ref.) | - | 0.149 |
|  | Group 2: LGE ≤4.2%, LV-GLS <14.3% | 1.32 (0.29–5.90) | 0.720 |  |
|  | Group 3: LGE >4.2%, LV-GLS ≥14.3% | 2.47 (0.63–9.61) | 0.193 |  |
|  | Group 4: LGE >4.2%, LV-GLS <14.3% | 4.29 (1.24–14.85) | 0.021 |  |
| Sex: Female (n=171) | Group 1: LGE ≤4.2%, LV-GLS ≥14.3% | 1 (ref.) | - |  |
|  | Group 2: LGE ≤4.2%, LV-GLS <14.3% | 1.83 (0.54–6.17) | 0.332 |  |
|  | Group 3: LGE >4.2%, LV-GLS ≥14.3% | 0.45 (0.08–2.50) | 0.364 |  |
|  | Group 4: LGE >4.2%, LV-GLS <14.3% | 3.29 (1.00–10.77) | 0.049 |  |
| Age ≤60 years (n=409) | Group 1: LGE ≤4.2%, LV-GLS ≥14.3% | 1 (ref.) | - | 0.187 |
|  | Group 2: LGE ≤4.2%, LV-GLS <14.3% | 0.45 (0.09–2.35) | 0.345 |  |
|  | Group 3: LGE >4.2%, LV-GLS ≥14.3% | 1.04 (0.31–3.51) | 0.944 |  |
|  | Group 4: LGE >4.2%, LV-GLS <14.3% | 1.54 (0.52–4.58) | 0.438 |  |
| Age >60 years (n=243) | Group 1: LGE ≤4.2%, LV-GLS ≥14.3% | 1 (ref.) | - |  |
|  | Group 2: LGE ≤4.2%, LV-GLS <14.3% | 2.98 (0.78–11.37) | 0.110 |  |
|  | Group 3: LGE >4.2%, LV-GLS ≥14.3% | 1.51 (0.30–7.49) | 0.615 |  |
|  | Group 4: LGE >4.2%, LV-GLS <14.3% | 7.26 (2.03–25.92) | 0.002 |  |
| Apical HCM (n=161) | Group 1: LGE ≤4.2%, LV-GLS ≥14.3% | 1 (ref.) | - | 0.151 |
|  | Group 2: LGE ≤4.2%, LV-GLS <14.3% | 0.72 (0.10–5.37) | 0.748 |  |
|  | Group 3: LGE >4.2%, LV-GLS ≥14.3% | - | - |  |
|  | Group 4: LGE >4.2%, LV-GLS <14.3% | 2.82 (0.48–16.66) | 0.253 |  |
| Non-apical HCM (n=491) | Group 1: LGE ≤4.2%, LV-GLS ≥14.3% | 1 (ref.) | - |  |
|  | Group 2: LGE ≤4.2%, LV-GLS <14.3% | 1.75 (0.60–5.13) | 0.304 |  |
|  | Group 3: LGE >4.2%, LV-GLS ≥14.3% | 1.36 (0.48–3.87) | 0.565 |  |
|  | Group 4: LGE >4.2%, LV-GLS <14.3% | 3.16 (1.24–8.05) | 0.016 |  |

^a^Adjusted for 5-year SCD risk score, LV ejection fraction, and LV apical aneurysm

CI, confidence interval; CV, cardiovascular; HR, hazard ratio; LGE, late gadolinium enhancement; LV, left ventricular; LV-GLS, left ventricular global longitudinal strain; SCD, sudden cardiac death

**Supplemental Figure 1. Cutoff values of LGE% and LV-GLS derived from maximally selected rank statistics.**

Maximally selected rank statistics identified 2.8% as the cutoff for LGE%, and 9.7% for LV-GLS.
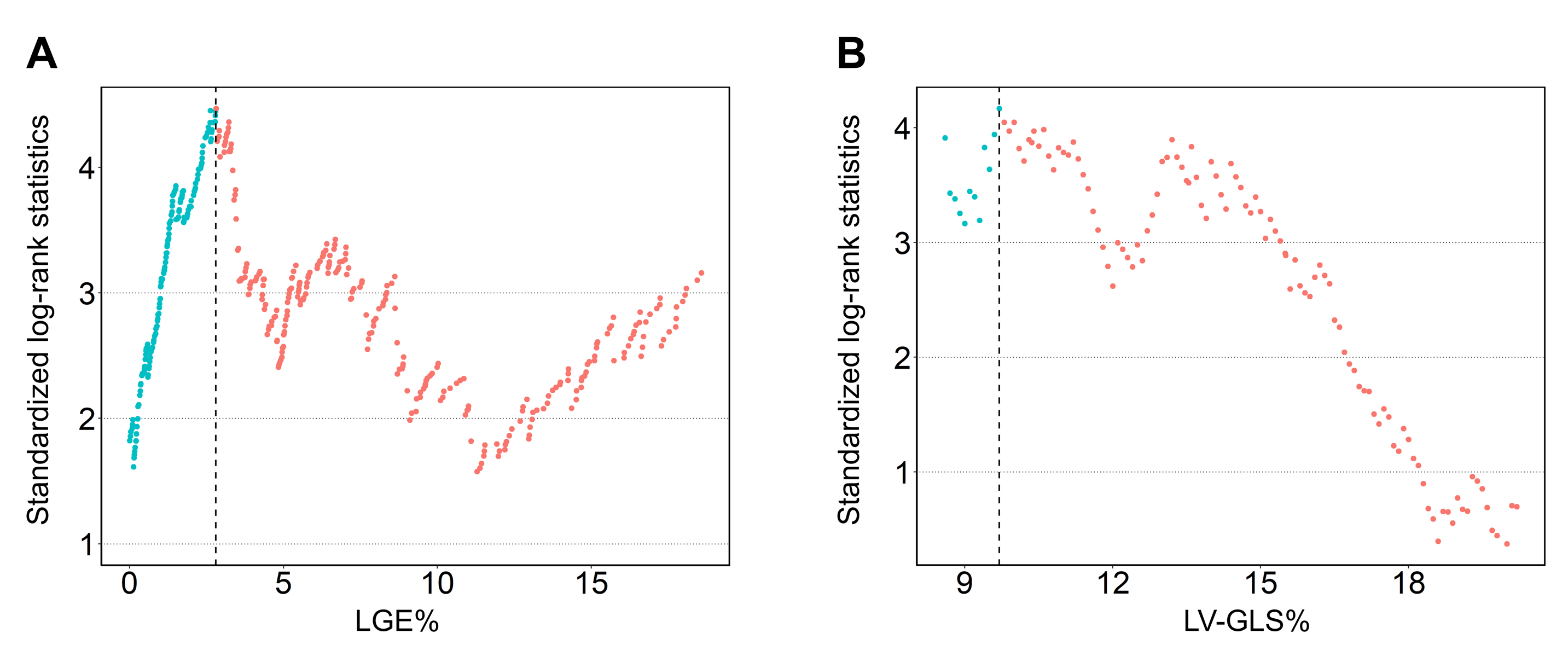


LGE, late gadolinium enhancement; LV-GLS, left ventricular global longitudinal strain

**Supplemental Figure 2. Sensitivity analysis in patients with preserved LV ejection fraction.**

Patients with an LV ejection fraction of ≥50% were included in the sensitivity analysis (n=630). When patients were divided using the same cutoffs from the entire cohort (LGE of 4.2% and LV-GLS of 14.3%), CV events were most frequent among those with both increased LGE (>4.2%) and decreased LV-GLS (<14.3%).


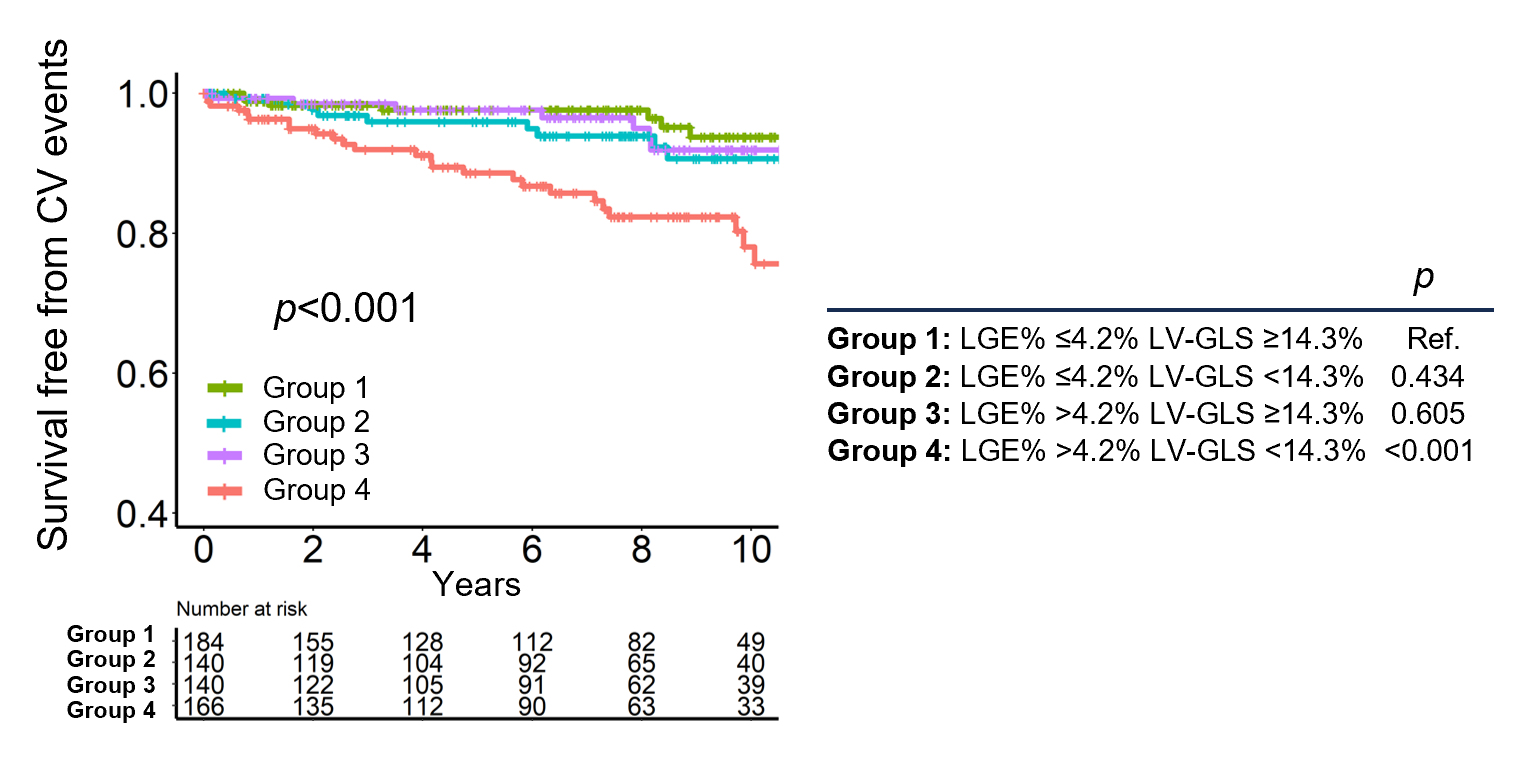


CV, cardiovascular; LGE, late gadolinium enhancement; LV, left ventricular; LV-GLS, left ventricular global longitudinal strain

**Supplemental Figure 3. Cumulative SCD-related events according to LGE% with 15% cutoff and LV-GLS.**

**(A)** Survival free from SCD-related events according to LGE% (≥15% vs <15%). **(B)** Survival free from SCD-related events according to four groups stratified by LGE% and LV-GLS.


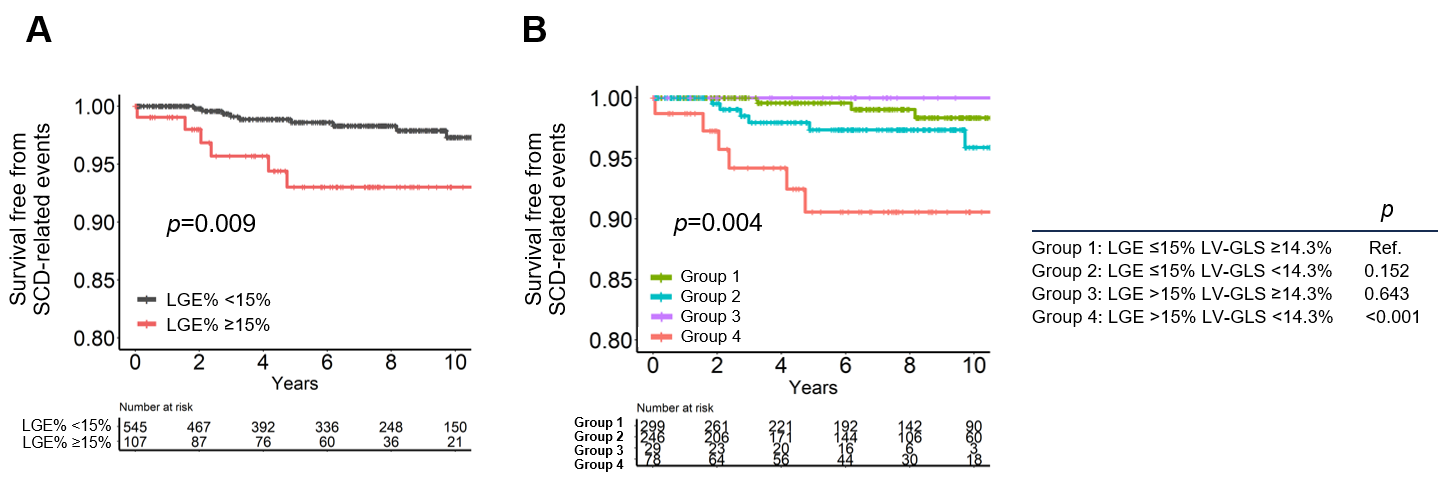

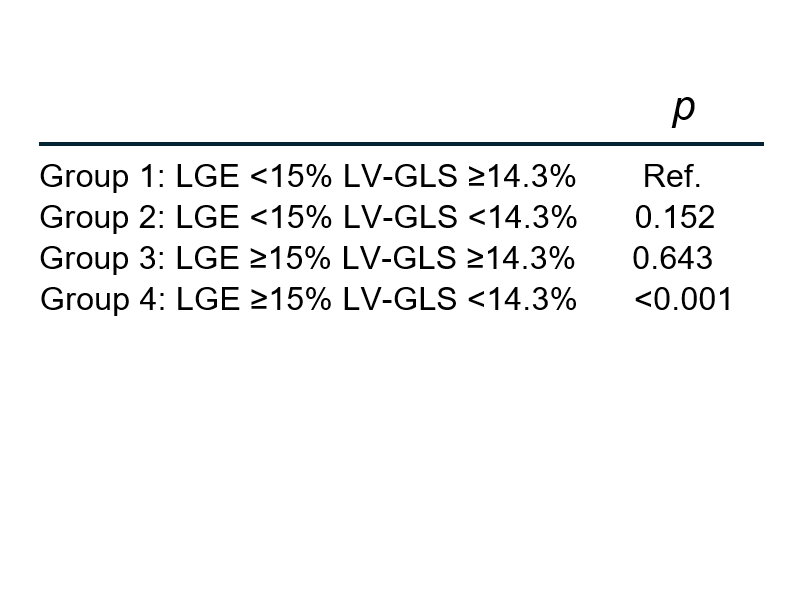


LGE, late gadolinium enhancement; LV-GLS, left ventricular global longitudinal strain; SCD, sudden cardiac death

**Supplemental Figure 4. Cumulative SCD-related events according to LGE% and LV-GLS cutoffs derived from the maximally selected rank statistics.**

(**A**) Survival free from SCD-related events according to LGE% (>2.8% vs. ≤2.8%). (**B**) Survival free from SCD-related events according to LV-GLS (<9.7% vs. ≥9.7%). (**C**) Survival free from SCD-related events according to LGE% and LV-GLS.


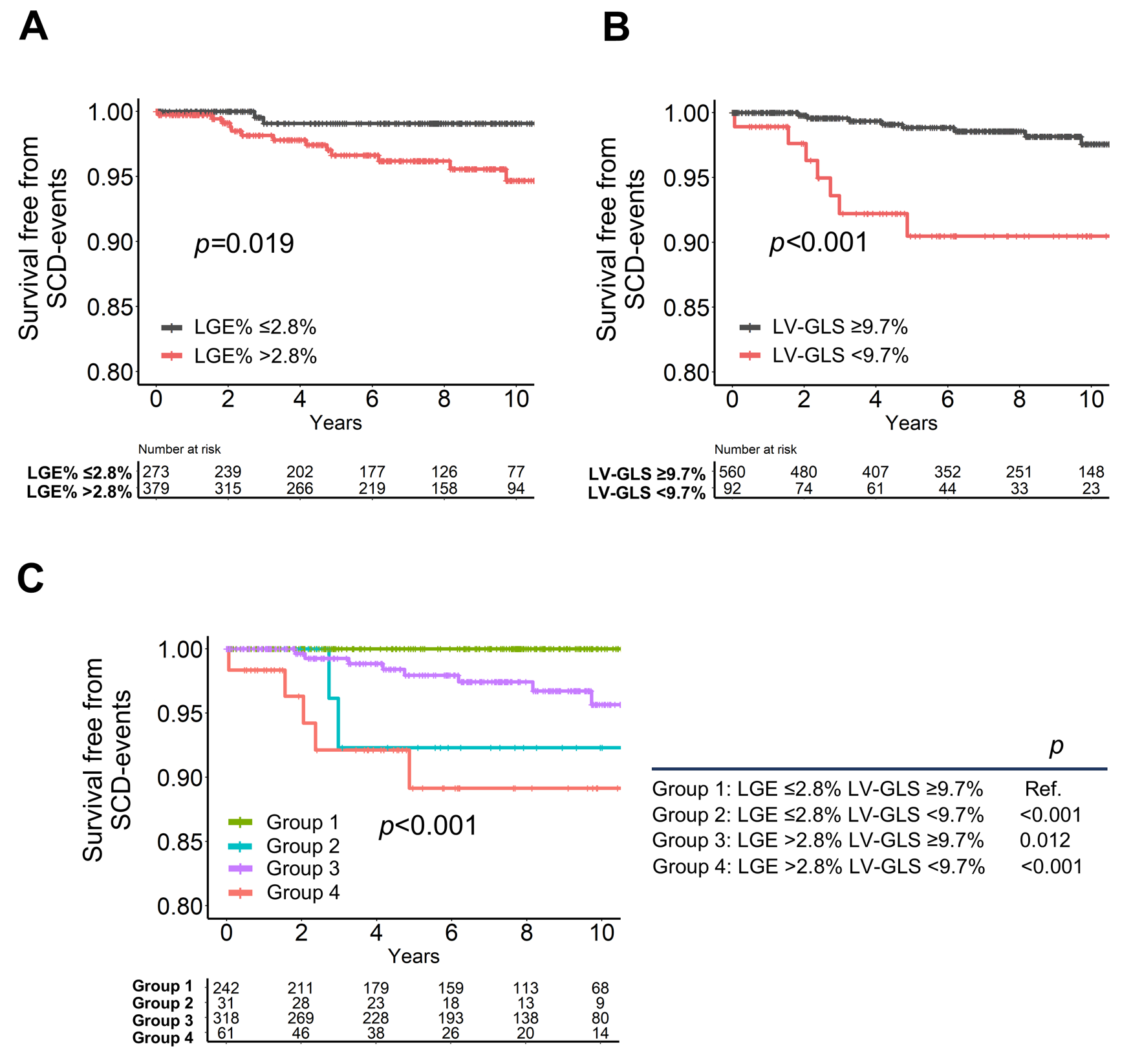


SCD, cardiovascular; LGE, late gadolinium enhancement; LV-GLS, left ventricular global longitudinal strain
